# Advanced Magnetic Resonance Imaging and Molecular Imaging of the Painful Knee

**DOI:** 10.1055/s-0043-1775741

**Published:** 2023-11-07

**Authors:** Jacob M. Mostert, Niels B.J. Dur, Xiufeng Li, Jutta M. Ellermann, Robert Hemke, Laurel Hales, Valentina Mazzoli, Feliks Kogan, James F. Griffith, Edwin H.G. Oei, Rianne A. van der Heijden

**Affiliations:** 1 Department of Radiology and Nuclear Medicine, Erasmus University Medical Center, Rotterdam, The Netherlands; 2 Department of Orthopedics and Sports Medicine, Erasmus University Medical Center, Rotterdam, The Netherlands; 3 Department of Radiology, Center for Magnetic Resonance Research (CMRR), University of Minnesota, Minneapolis, Minnesota; 4 Department of Radiology and Nuclear Medicine, Amsterdam University Medical Center, Amsterdam, The Netherlands; 5 Department of Radiology, Stanford University, Stanford, California; 6 Department of Imaging and Interventional Radiology Faculty of Medicine, The Chinese University of Hong Kong, Hong Kong; 7 Department of Radiology, University of Wisconsin-Madison, Madison, Wisconsin

**Keywords:** knee, pain, imaging, magnetic resonance imaging, positron emission tomography

## Abstract

Chronic knee pain is a common condition. Causes of knee pain include trauma, inflammation, and degeneration, but in many patients the pathophysiology remains unknown. Recent developments in advanced magnetic resonance imaging (MRI) techniques and molecular imaging facilitate more in-depth research focused on the pathophysiology of chronic musculoskeletal pain and more specifically inflammation. The forthcoming new insights can help develop better targeted treatment, and some imaging techniques may even serve as imaging biomarkers for predicting and assessing treatment response in the future. This review highlights the latest developments in perfusion MRI, diffusion MRI, and molecular imaging with positron emission tomography/MRI and their application in the painful knee. The primary focus is synovial inflammation, also known as synovitis. Bone perfusion and bone metabolism are also addressed.

Chronic knee pain is a very common and troublesome symptom across the population. Pain can be caused by many processes, such as trauma, inflammatory diseases, or degeneration. However, in a substantial number of patients, the cause of pain remains unknown. This dilemma can be largely attributed to our inability to accurately pinpoint the source of pain with current anatomy-based imaging approaches, leaving the relationship between knee pain and structural abnormalities far from fully understood. Recent developments in advanced magnetic resonance imaging (MRI) techniques and molecular imaging facilitate more in-depth research into the pathophysiology of musculoskeletal (MSK) chronic pain and, more specifically, inflammation. This research could lead to better targeted treatment and new imaging biomarkers for diagnosis or treatment response.

In the past few years, perfusion MRI, in particular dynamic contrast-enhanced (DCE)-MRI, has been extensively applied in research as a surrogate measure of inflammation. Inflamed tissues display hypervascularization due to the rapid formation of neovessels in response to the increased oxygen demand also known as neoangiogenesis. Most neovessels are structurally underdeveloped and lack pericytes to stabilize their vessel walls, leading to increased permeability. In addition, the release of inflammatory mediators also increases permeability.

DCE-MRI, also known as permeability imaging, is able to evaluate *perfusion,* which refers to flow within capillaries and venous sinusoids where transendothelial exchange occurs. Perfusion is increased in inflammatory diseases, such as rheumatoid arthritis (RA) and the childhood variant, juvenile idiopathic arthritis (JIA), that often manifests at the knee. In recent years, it was shown that inflammation also plays a critical role in osteoarthritis (OA), where inflammation of the joint lining, also known as synovitis, occurs. Increased perfusion also occurs during repair of damaged tissues, for instance around the bony defects seen in osteochondritis dissecans.

Other advanced techniques that evaluate perfusion parameters are arterial spin labeling (ASL) MRI and intravoxel incoherent motion (IVIM) MRI. The latter is a diffusion-based approach. More recently, diffusion MR and other MR approaches, such as double echo steady state (DESS) and double-inversion recovery (DIR), have been evaluated compared with contrast-enhanced (CE) MRI for assessment of inflammation. This would render the administration of contrast redundant, which certainly is an advantage in the pediatric population, but also in the adult population, given the recent new insights into the accumulation of gadolinium and also the increased costs of contrast agents.

The recent combination of positron emission tomography (PET) and MRI enables further exploration of knee pathophysiology by visualizing metabolic changes that may precede morphological or physiologic changes. Innovative radiotracers have also emerged, focusing on different cellular and molecular processes, thereby broadening the range of metabolic pathways that can be studied.

This review highlights the latest developments of perfusion MRI, diffusion MRI, and PET/MRI of the chronically painful knee. The primary focus is synovitis, as well as bone perfusion and metabolism. A brief overview of other applications is also provided to show the full potential of each imaging technique.

## Perfusion Imaging

### Dynamic Contrast-Enhanced Magnetic Resonance Imaging

#### Technical Perspective

DCE-MRI involves the intravenous injection of a gadolinium-based contrast agent (GBCA) followed by rapid acquisition of a time series of T1-weighted images. This method enables assessment of T1 changes over time that occur due to contrast accumulation and the corresponding T1 relaxation time shortening, which results in local signal enhancement. Several commercially available GBCAs have been used for DCE-MRI. Their relatively small molecular size allows them to diffuse from the vessel into the extravascular extracellular space, also known as interstitium. The rate at which GBCAs diffuse into the extravascular extracellular space is determined by blood flow, capillary permeability, capillary surface area, interstitial pressure, interstitial space, and interstitial flow. The gadolinium-induced T1-signal increase in a tissue of interest is therefore a composite measure of blood flow, perfusion, and interstitial diffusion.

DCE-MRI is usually based on T1-weighted three-dimensional (3D) spoiled gradient-echo sequences, where the exact sequence structure and pulse timing parameters depend on the specific application. 1 Choosing sequence parameters involves a tradeoff between coverage, spatial resolution, and temporal resolution. To image the knee adequately, a substantial field of view and spatial resolution are needed, while at the same time the temporal resolution needs to be adequate to capture fluctuations in contrast enhancement. 1 Implementation of parallel acceleration or undersampling techniques can significantly improve temporal resolution.

**Fig. 1:**
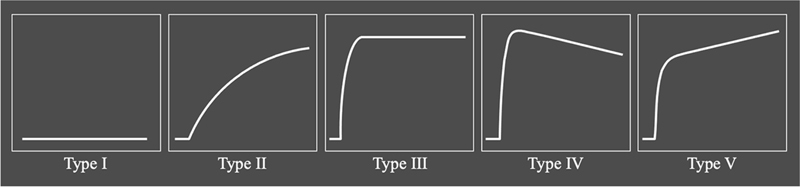
Time-intensity curve shape profiles. Type I: no enhancement. Corresponds to background voxels, nonenhancing bone, or necrotic tissue. Type II: slow progressive enhancement with no enhancement peak. Can be seen in skin, muscle, bone, and soft tissue boundaries or artifacts. Type III: rapid steep enhancement followed by plateau. Can be seen in inflamed synovium, muscle, and bone marrow edema. Type IV: rapid steep enhancement peak followed by a washout. Often located in arteries, veins, or highly perfused and severely inflamed tissue. Type V: rapid steep enhancement peak followed by progressive slow enhancement. May be seen in granulation tissue or fibrosis. (Reproduced with permission from Griffith JF, van der Heijden RA. Bone marrow MR perfusion imaging and potential for tumor evaluation. Skeletal Radiol 2023;52(3):477–491.) 91

GBCA-induced change in signal intensity over time within a tissue of interest can be visualized with time-intensity curves. Describing the shape of this time-intensity curve is a relatively straightforward method to characterize the underlying contrast behavior in vivo known as semiquantitative or *heuristic* analysis. A curve can be classified in several predefined shape types ( Fig. 1 ). In addition, several other semiquantitative parameters can be derived from the shape of the time-intensity curve, such as initial area under the curve, maximum (relative) enhancement, initial rate of enhancement, initial rate of washout, onset time of enhancement (T _onset_ ), and time to washout (T _washout_ ). Although semiquantitative analysis is relatively robust, the major downside is that semiquantitative parameters represent a mixture of circulatory and tissue properties and cannot be directly correlated to underlying physiology. 2 Another disadvantage is that between-subject differences related to contrast administration, such as bolus delay, and differences in acquisition are not considered. 3

Pharmacokinetic modeling can overcome these issues by implementing a mathematical representation of the assumed underlying vascular processes within a tissue of interest to quantify perfusion. It requires conversion of signal intensity to absolute contrast concentration, enabling harmonization across MR field strengths and comparison between subjects. 3 To convert signal to contrast concentration, acquisition of a native T1 map is needed before administration of the contrast agent, but T1 mapping can be sensitive to magnetic field inhomogeneity and artifacts related to flow. 4 Therefore, B1 mapping is typically also acquired.

Additionally, most pharmacokinetic models require an arterial input function (AIF) to be determined. The AIF represents the change in contrast concentration over time in an afferent blood vessel supplying the tissue of interest. The AIF can be extracted from the dynamic image by selecting a small number of voxels inside a supplying artery and taking the average signal intensity over time. Accurate AIF calculation requires a sufficient temporal resolution to be able to capture the peak of the first pass through the artery. This is where acceleration techniques might make a change by allowing good temporal resolution without compromising the spatial resolution. AIF estimation can also be influenced by flow artifacts, partial-volume effects, and nonlinear saturation effects of a high-contrast agent concentration. Attempts to achieve a more robust AIF estimation include groupwise AIF calculation by averaging across study participants, or using population-based average functions, but this can hide potentially important differences between subjects. 5

Several pharmacokinetic DCE-MRI models are available, and the most used model in MSK research is the model first reported by Tofts and Kermode. 6 The model yields parameters K ^trans^ , v _e_ , and k _ep_ . K ^trans^ represents the volume transfer between blood plasma and the extracellular extravascular space, mainly influenced by flow and vascular permeability. The fractional volume of the extravascular extracellular space (v _e_ ) represents the interstitial space volume. The exchange rate constant between the extracellular extravascular space and blood plasma, k _ep_ , is given by k _ep_ = K ^trans^ /v _e_ and thus is not an independent parameter. The extended Tofts model additionally incorporates the fractional volume of blood plasma v _p_ and is most relevant in highly vascularized tissues. 7

One of the main barriers limiting more widespread clinical implementation of DCE-MRI and other advanced quantitative methods is the lack of reproducible methods. A first step toward better reproducibility is standardization of both the acquisition and analysis.

#### Clinical Applications

The main focus of DCE-MRI and also the only established clinical application has been tumor imaging, in which DCE-MRI is used for tumor characterization, differentiation between benign and malignant lesions, evaluation of treatment response, and assessment of postsurgical recurrence. Beyond oncologic imaging, DCE-MRI has the potential to assess inflammatory joint disease. In the knee, OA, RA, and JIA all have inflammatory components in which DCE-MRI can be useful.

##### Osteoarthritis

We now know that OA is not simply a wear-and-tear disease of the cartilage, but that inflammation plays a critical role in its development and progression. Consequently, disease-modifying OA drugs (DMOADs) targeting reduction of inflammation are being developed. Synovitis is the main inflammatory feature and thus an important focus in clinical trials. Static contrast-enhanced MRI is the gold standard for assessment of synovitis, but DCE-MRI is suggested to be more sensitive. Enhanced synovium can also include more chronic fibrous regions, next to active inflammation. By providing temporal uptake information, it is possible to differ between regions with active inflammation with more pronounced early contrast uptake and fibrotic regions with a slower uptake profile that keeps accumulating.

**Fig. 2:**
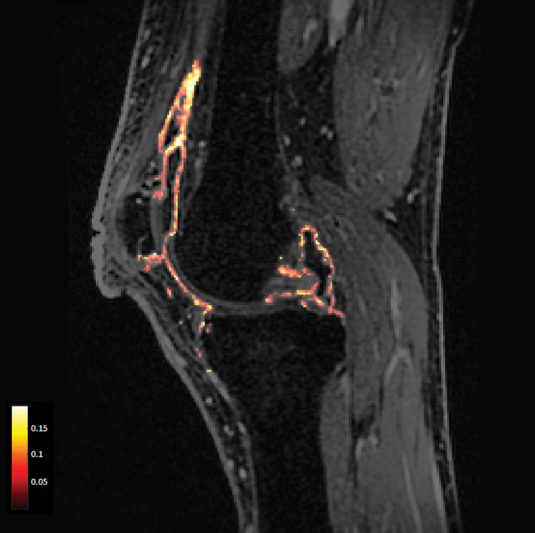
Sagittal magnetic resonance dynamic contrast-enhanced magnetic resonance image with a map of K ^trans^ values within the synovium of the knee in a patient with knee osteoarthritis.

Fig. 2 shows an example of a K ^trans^ -map of the knee synovium in an OA patient. A meta-analysis demonstrated a positive correlation for both CE-MRI and DCE-MRI between macroscopic and microscopic features of synovitis, with DCE-MRI showing the highest pooled correlation coefficients. 8 In addition, a strong correlation was found between DCE-MRI parameters for synovitis and knee pain symptoms, with some DCE parameters even showing a stronger correlation with the Knee injury and Osteoarthritis Outcome Scores (KOOS) than CE-MRI. 9 Other structures of interest that might be the source of pain in OA are the infrapatellar fat pad and bone marrow lesions (BMLs). In both the infrapatellar fat pad 10 and BMLs, 11 an association has been demonstrated between knee pain and DCE-MRI parameters of the respective structure.

As previously mentioned, one of the main challenges for widespread implementation of (quantitative) DCE-MRI is reproducibility. MacKay et al studied test-retest repeatability and sensitivity to change of DCE-MRI parameters of the synovium in patients with knee OA. With semiautomatic segmentation of the synovium and pharmacokinetic modeling, the parameter K ^trans^ yielded the best repeatability (intraclass correlation: 0.90) and sensitivity to change, indicating that K ^trans^ is likely to be the optimal DCE-MRI biomarker to use. 12

DCE-MRI of the synovium was recently used to evaluate treatment response in multiple studies. Weight loss 13 and exercise therapy 14 did not result in a significant change in DCE-MRI metrics despite a decrease in pain symptoms. However, there was also no significant change in other measures of inflammation, such as synovitis. This indicates that these treatments do reduce pain but not through reduction of synovial inflammation.

For intra-articular corticosteroid injection, mixed results were demonstrated. 15 16 The randomized controlled trial by Riis et al reported no significant effect of steroids and no change in K ^trans.^ . The KOOS pain score was weakly correlated with CE-MRI and not with DCE-MRI. 15 Gait et al demonstrated the opposite, namely a significant change in pain and K ^trans^ and a stronger correlation with DCE-MRI-derived measures than CE-MRI. 16 DMOADs specifically targeting inflammation might have a bigger impact. DCE-MRI can play a role in patient selection and clinical evaluation of these drugs.

##### Rheumatoid Arthritis

In RA of the knee, inflammation affects the synovium, subchondral bone, and intra-articular and extra-articular fat tissue, leading to subsequent cartilage loss, bone erosion and subchondral cysts, destabilization and joint malalignment, especially when untreated. DCE-MRI perfusion parameters are strongly correlated with indicators of RA-induced inflammation in histologic biopsies. 17 DCE-MRI of the knee has good reproducibility, 18 and with pharmacokinetic modeling, it could be used to detect and quantify synovial inflammation in patients with early inflammatory arthritis 19 and potentially differentiate between RA and other types of inflammatory arthritis. 20

##### Juvenile Idiopathic Arthritis

Disease activity in children with JIA of the knee has been studied with DCE-MRI. Semiquantitative DCE-MRI parameters differentiated between active and inactive JIA, but the quantitative parameters did not. 21 This was potentially due to the susceptibility of these parameters to errors in the calculation of the inputs. Semiquantitative DCE-MRI parameters also showed substantial correlation with static MRI scores of disease activity. 22 In a study by Workie et al, quantitative DCE-MRI parameters showed excellent correlation with clinical assessments. 23 This finding indicates that semiquantitative DCE-MRI has the potential to be used to monitor disease activity and treatment effect. For quantitative DCE, the results are mixed, but as previously mentioned, the acquisition and analysis method could have had a major impact, and standardization is needed.

### Arterial Spin Labeling Magnetic Resonance Imaging

#### Technical Perspective

In contrast to other imaging methods, ASL imaging uses endogenous blood water as an intrinsic agent to measure tissue perfusion. 24 As a nonradioactive, non–contrast-enhanced and noninvasive approach, ASL imaging eliminates concerns regarding exposure to ionizing radiation or the potential toxicity of exogenous contrast agents when applied to human research studies. ASL may be well suited for longitudinal monitoring of disease progression and routine evaluation of therapy response, particularly in patients with renal deficiency and in children.

The feasibility of bone marrow ASL imaging was demonstrated in the vertebrae that are mainly composed of red bone marrow in young and middle-aged patients. 25 ASL imaging can also be used to measure knee epiphyseal bone marrow blood flow (BMBF) and reflect disease-related changes in juvenile osteochondritis dissecans (JOCD) patients. 26 27 However, these studies also highlighted several intrinsic challenges. First, the inherent low signal-to-noise ratio (SNR) of ASL imaging is exacerbated by inherent low BMBF. Second, the complex vascular arrangement of knee arteries, along with low blood flow velocity, makes it difficult to use higher SNR ASL imaging methods, such as pseudo-continuous ASL approaches. 28

Additional methodological limitations also impact the quality and coverage of the ASL acquisitions. First, the previously used single-shot fast spin-echo image readout, although robust, only supports single-slice coverage. Second, readout-segmented echo planar imaging (RESOLVE), a technique insensitive to static magnetic field (B _0_ ) inhomogeneity and susceptibility, although advantageous, requires unacceptably long acquisition times with low spatial and temporal SNR as well as low spatial SNR efficiency (∼ 15 minutes, three or more segments for satisfactory image quality) 29 and therefore is impractical, especially for children, older adult patients, and bilateral knee studies.

ASL imaging at ultra-high field (UHF), such as 7 T, benefits from increased SNR, prolonged blood and tissue T _1_ , and improved parallel imaging performance that helps overcome the previously mentioned intrinsic challenges and methodological limitations by reducing ASL imaging acquisition times. However, these benefits can only be realized after existing technical challenges of 7-T imaging have been addressed, including deteriorated susceptibility-induced B _0_ inhomogeneity, elevated physiologic noise, increased motion sensitivity, and the constructive and destructive interferences of radiofrequency (RF) fields. 30 Such interferences result in significant spatial variations in the transmit B _1_ (B _1_^+^ ) fields dependent on an individual's body geometry and tissue distribution.

Additional constraints at UHF MRI is the inhomogeneity in the electric fields responsible for limitations in power deposition due to concerns about local heating. Recent preliminary knee ASL imaging studies on 7 T demonstrated that the current clinically approved single-transmit MRI system and associated imaging methods are incapable of managing the B _1_^+^ fields needed to realize the promised improvement in imaging quality, reliability, and robustness of 7 T while existing RF coils are unable to provide adequate B _1_^+^ coverage for optimal ASL imaging.

#### Clinical Applications

ASL is a relatively new technique that has been applied in oncology research and muscle perfusion studies. With regard to the knee, the main focus has been JOCD, a disease of the immature skeleton that affects children's knees and may lead to loose bodies in the joint.

Bone blood flow and perfusion are vital to both bone development via endochondral ossification and bone health throughout life. Angiogenesis plays a pivotal role in endochondral ossification (i.e., cartilage to bone transformation), growing bone, bringing cells and nutrients necessary to form new bone and bone marrow. 31 Any disruption to the angiogenesis process can significantly impact bone development, growth, and repair. Blood supply is also necessary for bone remodeling, the process of bone resorption and formation that maintains the integrity of the skeletal system throughout life. Conditions that impair bone blood flow can lead to significant bone health issues, such as fractures, impaired healing, decreased bone density, and osteonecrosis. Thus bone blood flow and perfusion are important physiologic parameters of bone vascularity and viability. Assessment and longitudinal monitoring of bone perfusion can provide valuable insights into bone health and associated diseases.

**Fig. 3:**
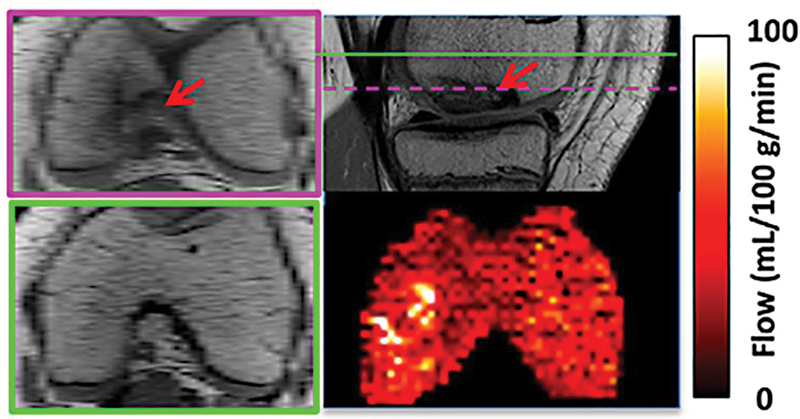
Anatomical images and bone marrow blood flow maps from a patient with juvenile osteochondritis dissecans. Green lines indicate the slice positions for arterial spin labeling images and corresponding anatomical images, and pink lines indicate distal anatomical images showing lesions.

In the past, different imaging modalities were applied for in vivo evaluation of bone perfusion, including PET, DCE-MRI, and static bone scintigraphy. Specifically, static bone scintigraphy was shown to have great diagnostic and prognostic potential for the diagnosis and prognosis of JOCD. Whereas other diagnostic modalities, such as MRI or radiographs alone, lack accuracy in predicting healing in JOCD patients, bone blood flow measurements and radiographs together dramatically improve diagnostic accuracy. 32 In addition, relative blood flow changes between the focal lesion and the surrounding healthy bone are highly correlated with clinical outcomes. 33 However, because of concerns of exposure to ionizing radiation and the potential toxicity of exogenous contrast agents, the application of these methods for assessing bone perfusion is limited. ASL could potentially solve these issues by allowing noninvasive exogenous contrast-free perfusion imaging ( Fig. 3 ).

Preliminary studies demonstrated the feasibility of ASL imaging for bone perfusion at 3 T and 7 T that can detect bone marrow perfusion changes in clinically relevant disease. 26 27 However, further technical development and research are needed to overcome current challenges and enhance future clinical application.

## Diffusion Imaging

### Diffusion-weighted Imaging

#### Technical Perspective

Diffusion-weighted imaging (DWI) is an MRI technique that uses the random motion of water molecules in biological tissues to provide information about tissue microstructure. Water molecules diffuse differently depending on the barriers they encounter, such as cell membranes and fibers. The extent of water diffusion can be quantified using the apparent diffusion coefficient (ADC), a measure of the rate of diffusion in a specific region of interest.

DWI is achieved by applying motion-probing gradients (MPGs) in different directions before and after a 180-degree RF pulse. This creates contrast between regions with different diffusion properties, with moving water molecules experiencing signal attenuation and stationary water molecules experiencing signal enhancement. Based on this signal attenuation, ADC values are generated on a pixel-by-pixel basis and can be visualized as parametric maps. ADC maps, combined with appropriate biophysical modeling, can provide information about tissue microstructure, cellularity, and water content. Higher ADC values indicate more freely diffusing water molecules, whereas lower values indicate restricted or hindered diffusion.

The b-values refer to the strength of the MPGs used during DWI image acquisition. They are quadratically proportional to the strength of the MPGs and expressed in units of seconds per square millimeter. The b-value used in the DWI experiment affects its sensitivity to different types of diffusion, such as free diffusion, restricted diffusion, or pseudo-diffusion, due to microcirculation. Lower b-values make DWI more sensitive to microcirculation, resulting in “fast diffusion.” Higher b-values make DWI more sensitive to restricted diffusion due to barriers such as cell membranes and fibers, resulting in “slow diffusion.” Varying the b-values can separate the different types of diffusion, allowing more information about the underlying tissue microstructure to be obtained. In MSK DWI protocols, b-values up to 800 s/mm ^2^ should be obtained (ideally 50, 400, and 800). 34 35

DWI can differentiate tissues and lesions based on their diffusion displacements. In the human body, biological tissues hinder diffusion due to their internal architecture, such as cell membranes and macromolecules, called “restriction of diffusion” or anisotropic diffusion. DWI visualizes slow or restricted diffusion regions as hyperintense and fast diffusion regions as hypointense. This ability to differentiate tissues and lesions using their diffusion properties enhances the capability of conventional MRI, allowing radiologists to recognize, characterize, and diagnose specific entities.

#### Clinical Applications

DWI is increasingly popular for the diagnostic strategy and assessment of therapeutic response of bone and soft tissue lesions, where it aids in characterizing lesions, guiding biopsy, determining tumor grading, and tracking tumor response to treatment. However, DWI has limitations because values for benign and malignant masses can overlap. DWI has also been applied in assessing the presence and severity of sacroiliitis as well as articular cartilage integrity.

##### Rheumatoid Arthritis

Some research has been performed in RA patients using DWI, but most studies focused on the hand, wrist, and craniocervical junction and not on the knee. Some studies have been performed using DWI to gauge the presence of synovitis in wrist and hand arthritis in RA patients and compared it with contrast-enhanced MRI as the reference standard, 36 37 showing a sensitivity of 75.6% and a specificity of 89.3% for the detection of synovitis using DWI. 36 However, ADC values did not significantly decrease over 6 months, whereas qualitative MRI and clinical parameters showed improvement in craniocervical synovial joints in early RA patients. 38

##### Juvenile Idiopathic Arthritis

**Fig. 4:**
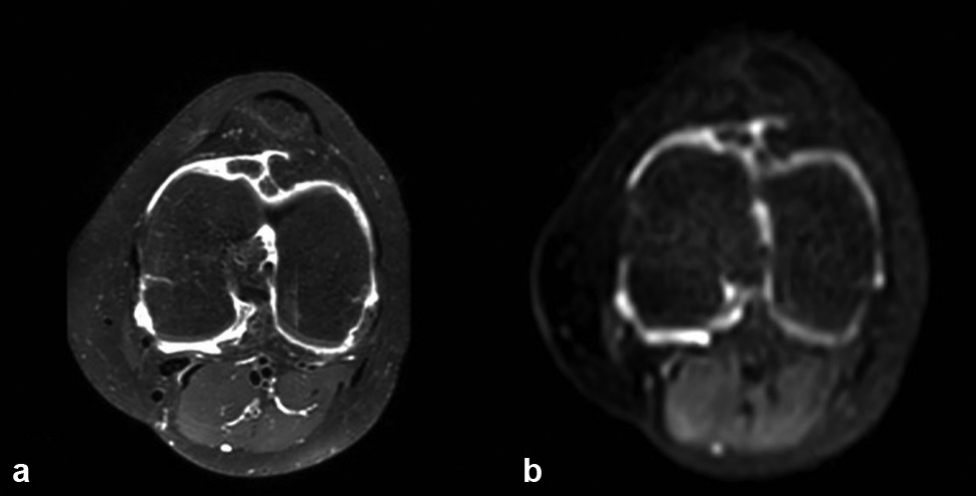
Anatomical and diffusion imaging of the knee in an 11-year-old female patient with extended oligoarticular juvenile idiopathic arthritis. ( **a** ) T1-weighted fast spin-echo magnetic resonance imaging after gadolinium-based contrast agent injection. ( **b** ) Diffusion-weighted imaging.

DWI has been studied in JIA affecting the knee and sacroiliac (SI) joints. ADC values are commonly higher in the SI joints of patients with enthesis-related arthritis than in control subjects with mechanical lower back pain. 39 ADC values reduced more in radiologic responders with anti-tumor necrosis factor treatment than in radiologic nonresponders. 40 DWI can be used in the knee joint in JIA to differentiate between synovial proliferation and joint fluid. 41 42 Fig. 4 shows an example.

### Diffusion Tensor Imaging

#### Technical Perspective

The unique structure of different tissues imposes directional restrictions on fluid diffusion, introducing a spatial orientation dependence in diffusion known as anisotropy. Diffusion tensor imaging (DTI) combines multiple DWI scans acquired using multiple gradient directions to identify directionality of diffusion. A minimum of seven DWI scans (one b = 0 and six different directions) is required but usually does not provide sufficient SNR for reliable estimates of DTI parameters. Acquiring multiple averages or additional directions can help improve DTI robustness and accuracy at the cost of increased scan time. Mean diffusivity (MD) and fractional anisotropy (FA) are commonly reported DTI measures. MD is a measure of average diffusion over all directions and sensitive to changes in fluid diffusion. FA is a measure of the amount of anisotropy and sensitive to changes in tissue microstructure.

Low SNR is a frequent problem with DTI. Signal attenuation due to diffusion in already signal starved tissues, and distortion and blurring artifacts inherent in echo planar imaging (EPI) trajectories reduce the accuracy and robustness of DTI estimates and require a multistep analysis pipeline that includes denoising, image registration, and registration to a non-EPI scan. Several toolboxes have been developed for analyzing DTI data; however, they are designed for brain or cardiac imaging and not optimized for the knee, which contains small structures that can be difficult to resolve due to magnetic field inhomogeneities and short-T2 tissues with low signal. Thus the user must check the performance of such tools at each step in the analysis pipeline to ensure accurate DTI measurements.

#### Clinical Applications

DTI has been applied in MSK research to study muscle structure around the knee, knee joint articular cartilage, and synovitis.

##### Osteoarthritis/Rheumatoid Arthritis

Synovitis is often associated with and can contribute to the progression of various types of arthritis including OA and RA. The intensity and extent of synovitis are determined with invasive methods such as histologic examination and DCE-MRI. DTI has been proposed as a noninvasive way to measure synovial inflammation, and its sensitivity has been repeatedly demonstrated. 43 44 However, the biological underpinnings of the DTI signal in the synovium are still unclear.

**Fig. 5:**
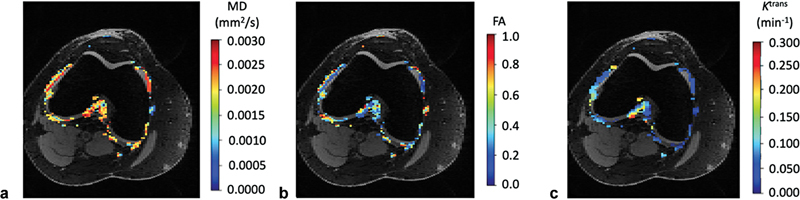
Sample measurements of ( **a** ) mean diffusivity (MD), ( **b** ) fractional anisotropy (FA), and ( **c** ) K ^trans^ within the synovium. Average MD had a significant positive correlation with average K ^trans^ ; average FA had a significant negative correlation with average K ^trans^ . (Adapted with permission from Sandford HJ, MacKay JW, Watkins LE, Gold GE, Kogan F, Mazzoli V. Gadolinium-free assessment of synovitis using diffusion tensor imaging. NMR Biomed 2022;35(1):e4614.) 43

Sandford et al demonstrated significant negative correlations in OA patients between average FA and K ^trans^ and the MRI OA knee score (MOAKS) total knee synovitis score, and significant positive correlations between MD and K ^trans^ and MOAKS ( Fig. 5 ). 43 However, Agarwal et al showed a significant increase in FA in RA patients compared with age-matched healthy controls and showed the level of inflammatory cytokines in the synovial fluid correlated positively with FA and negatively with MD. 45 Tripathi et al tested patients with various types of arthritis including OA and RA, and they showed that overall, FA in the synovium is positively correlated with multiple cellular inflammatory markers of inflammation and the Rooney Synovitis Score. 44 DTI measures are sensitive to synovitis, but more work is needed to understand the mechanisms behind their relationship and determine their sensitivity to change in the degree of synovitis and their diagnostic value compared with gold standard GBCA methods.

### Intravoxel Incoherent Motion

#### Technical Perspective

IVIM is a method of separating out different types of fluid motion within a voxel in DWI scans. Due to the random orientation and small scale of capillaries, perfusion has an effect similar to random Gaussian diffusion on DWI. 46 Both this pseudo-diffusion and diffusion result in a separate monoexponential decay as a function of b-value. The perfusion (pseudo-diffusion) component decays significantly faster than the diffusion component, allowing these two signals to be separated by fitting DWI data acquired with multiple b-values to the IVIM equation 47 :



Where f is the fraction of the attenuation due to perfusion, D* is the pseudo-diffusion coefficient, and D is the diffusion coefficient. In other words, IVIM can distinguish between perfusion and diffusion, and the parameters f and D* are sensitive to changes in tissue perfusion. IVIM is particularly useful in areas of tissue inflammation where it is necessary to separate edematous inflamed tissue from surrounding fluid and where changes in tissue perfusion may indicate disease activity. Although IVIM in the knee is a promising method, more work is needed to determine the optimal parameters for routine use.

IVIM analysis is plagued by many of the same issues as DTI analysis including low signal, blurring and distortion artifacts, high computational burden, and analysis toolboxes that are not optimized for MSK data. However, several toolboxes are available that do perform adequately with user supervision, and there is promise that much of this analysis can be performed through machine learning, although this has not yet been tested with respect to knee imaging.

#### Clinical Applications

In past years, IVIM was applied in MSK research to study knee soft tissue tumors, muscle perfusion, and synovitis.

**Fig. 6:**
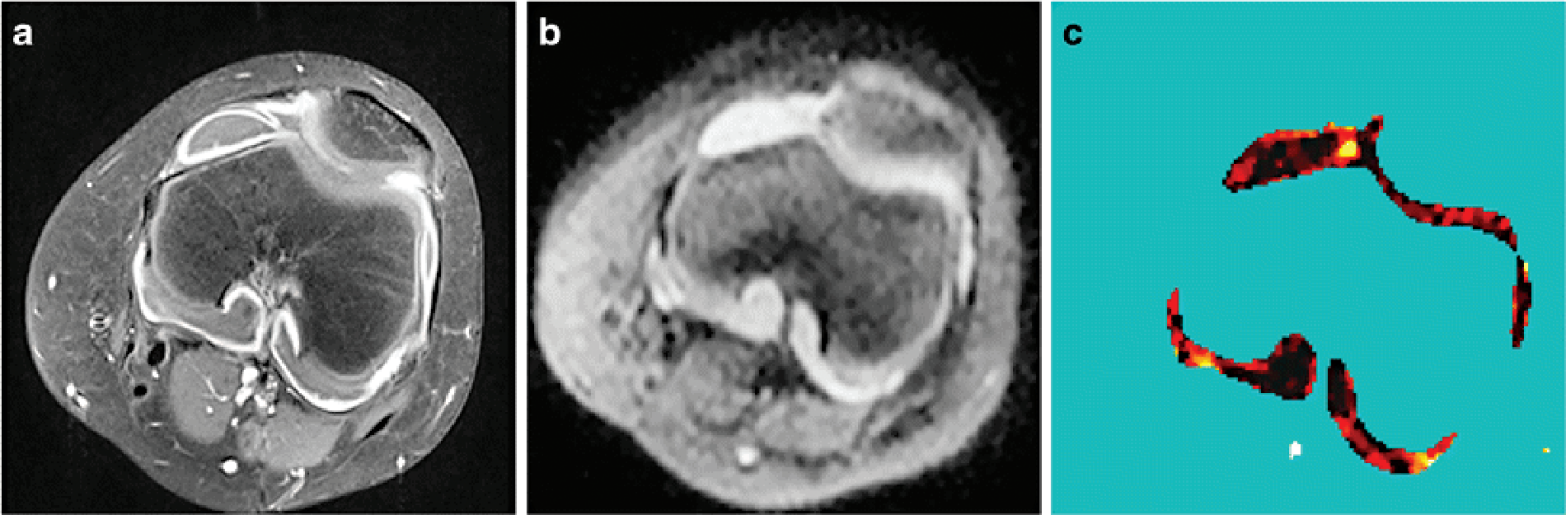
( **a** ) Contrast-enhanced magnetic resonance imaging: Synovial hypertrophy and enhancement is visible. ( **b** ) Diffusion-weighted imaging: There is poor delineation between the synovium and effusion due to T2 shine-through. ( **c** ) f-map: Improved delineation between synovium and effusion. (Reproduced with permission from Hilbert F, Holl-Wieden A, Sauer A, Köstler H, Neubauer H. Intravoxel incoherent motion magnetic resonance imaging of the knee joint in children with juvenile idiopathic arthritis. Pediatr Radiol 2017;47:681–690.) 49

Partial-volume effects and T2 shine-through effects (where small amounts of effusion appear as inflammation) make it difficult to differentiate between synovial perfusion and joint effusion on non-CE images. IVIM is able to separate these signals. Introductory studies have demonstrated that IVIM parameter maps were more effective than DWI images at differentiating inflamed synovium and effusion in patients with JIA as shown in Fig. 6 . 48 49 Future work is needed to determine how this method compares diagnostically with gold standard measures, but IVIM shows promise as a way to identify regions of synovitis.

Treatment of meniscal disorders occasionally involves CE-MRI because knowledge of parameniscal perfusion is relevant to treatment planning. Guo et al demonstrated that the IVIM parameters f and D* can provide noninvasive perfusion measures to distinguish the red and white meniscal regions and detect microcirculation changes in the disordered meniscus. 50 However, more work is needed to determine the limits of this method and evaluate how it compares with CE-MRI.

## Other Noncontrast-Enhanced Sequences

### Double-echo in Steady-state

Quantitative double echo in steady state (qDESS) MRI is a relatively recent technique that can be applied to visualize multiple sources of pain in the knee. It is a 3D gradient-spoiled steady-state sequence that acquires an echo before and after a spoiler gradient. The acquired images can be assessed either separately or combined into one image. In the context of inflammation, a promising application of qDESS is the assessment of synovitis without the need for a contrast agent, making use of its DWI capability. The separation of synovial tissue and joint fluid is further enhanced by increasing the magnitude of the spoiler gradient between the two echoes. Using image processing, specifically to null the signal intensity from joint fluid, the delineation of synovium can be further enhanced. 51

**Fig. 7:**
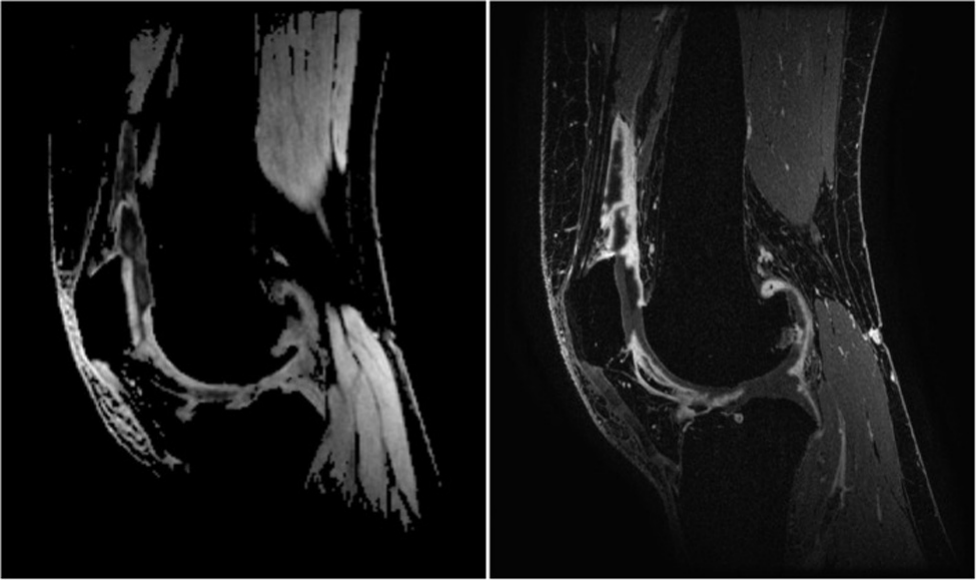
Sagittal quantitative double echo in steady state (qDESS) hybrid difference image (left) and contrast-enhanced magnetic resonance imaging (right), both at the level of the patella. (Reproduced from de Vries BA, Breda SJ, Sveinsson B, et al. Detection of knee synovitis using non-contrast-enhanced qDESS compared with contrast-enhanced MRI. Arthritis Res Ther 2021;23(1):1–9.)

In a validation study of 30 patients with varying degrees of knee OA by de Vries et al, qDESS was compared with static CE-MRI as the reference standard. Using semiquantitative grading at different locations in the knee, it was found that synovitis sum scores on qDESS and CE-MRI correlated very strongly and that qDESS had a high sensitivity (1.00) and specificity (0.91) for detecting synovitis of a mild degree or higher. However, it was also shown that the severity of synovitis was systematically underrated with qDESS compared with CE-MRI ( Fig. 7 ). 52

Other applications of qDESS are morphological knee assessment in a short scan time. Semiquantitative grading of knee OA can be performed reliably for most features, although it is slightly less sensitive for BML detection. Finally, qDESS can be used to perform morphometric evaluation of the articular cartilage as well as quantitative assessment of cartilage and meniscus composition using T2 relaxometry. Because morphological assessment and T2 mapping can be performed concurrently within 5 minutes, qDESS has the potential to be an efficient method of analyzing knee pain in OA. 53

### Double-Inversion Recovery

DIR MRI is a relatively recent technique that also shows promise at assessing synovitis without the need for a contrast agent. An inversion recovery sequence can void the signal of a single tissue. DIR is an inversion recovery variant that uses two nonselective 180-degree inverting pulses instead of one, resulting in suppression of two tissues. Pilot studies have shown the promise of this technique in the assessment of synovitis by suppressing both joint effusion and fat tissue. 54 A recent study by Verkuil et al demonstrated that DIR MRI adequately delineated the knee synovium in children with JIA and enabled synovial thickness measurement similar to that of CE-MRI. 55 One of the challenges of DIR is to time the two 180-degree RF pulses so the inverted longitudinal magnetization of fat and fluid reaches the null point concurrently when image acquisition occurs.

### Positron Emission Tomography/Magnetic Resonance Imaging

#### Technical Perspective

Although advanced MRI techniques enable the assessment of knee structure and biochemical composition, techniques like perfusion and diffusion imaging are not yet widely implemented in clinical practice. PET imaging has been implemented in clinical practice for years, enabling metabolic evaluation. This is crucial for studying disease pathogenesis and treatment response because metabolic changes may precede structural or even biochemical changes in MSK disease. 56 PET can be used to evaluate metabolic parameters on a cellular or even molecular level. PET imaging requires intravenous administration of a radiopharmaceutical tracer and allows metabolic activity to be assessed quantitatively. The recent introduction of integrated PET and MRI enables high-resolution anatomical imaging and functional imaging coupled with perfectly coregistered metabolic imaging, opening many new opportunities in both research and in clinical applications, especially for nononcologic applications.

In the context of MSK imaging, two commercially available PET tracers are most commonly used. Fluorine-18 labeled fluoro-2-deoxy-2-D-glucose ( ^18^ F-FDG) is a glucose analog, showing areas of increased glucose metabolism. It is extensively used in oncology to detect metastases or tumor recurrence and to monitor response to therapy. However, ^18^ F-FDG uptake can also indicate acute-phase cellular response areas such as inflammation, and it has proven useful in identifying increased glucose consumption in cases of MSK inflammation and infection. 57 Another tracer, ^18^ F-sodium fluoride ( ^18^ F-NaF), is a bone-seeking agent, mainly absorbed at newly mineralized bone sites, and provides a marker of bone metabolism and remodeling. 58 59 There are also, in addition, several promising new tracers, some of which may be useful for studying the origin of pain or inflammation.

Analysis of PET/MR images in a clinical setting is usually done by visual assessment with identification of areas of higher tracer accumulation, combined with anatomical information from corresponding morphological images. Because PET is a quantitative technique, visual assessment can be supported by quantification of tracer accumulation in tissues of interest. The metric most often used for this purpose is the standardized uptake value (SUV), given by the measured activity concentration in a volume of interest normalized to total injected activity and the patient's body weight. Mean and maximum SUV (SUVmean and SUVmax) measures allow for semiquantitative evaluation of tracer uptake with low precision error. 60

The use of dynamic PET and pharmacokinetic modeling of tracer uptake offers a fully quantitative approach to PET image analysis. 61 But it does require acquisition of dynamic PET data, typically ≥ 45 minutes, which are then reconstructed into shorter time frames to generate an AIF and a time activity curve. 62 By fitting a three-compartment model, 61 parameters describing the movement of PET tracer between blood plasma, extravascular bone, and bone mineral compartments can be estimated: for instance, *K*_1_ (transit rate of [ ^18^ F]NaF from plasma to the extravascular bone compartment, i.e., bone perfusion), the extraction fraction (the fraction of [ ^18^ F]NaF entering bone tissue and binding to bone matrix as opposed to being cleared into the plasma), and *K _i_* (clearance rate of [ ^18^ F]NaF from the blood plasma to the bone mineral compartment). Dynamic PET with ^18^ F-NaF is sensitive to variations in bone vascularization and metabolism in the knee joint among different knee tissue types. 63

Despite its many advantages, clinical adaptation of PET/MRI for MSK applications poses several challenges, such as MR-based attenuation correction in the vicinity of bone or metal implants, lack of standardization for quantitative analysis outside tumor lesions, relatively long image acquisition times, and limited availability of dedicated PET/MR machines.

#### Clinical Applications

Adaptation of PET for MSK applications has increased over the past 20 years and continues to grow. Although the main indication for PET remains oncologic imaging, the application of PET/MRI in nononcologic MSK disease has increased in several research domains and clinical applications, including the assessment of OA, RA, and the detection of pain generators.

##### Osteoarthritis

PET/MRI of the painful knee has largely focused on OA, with PET/MRI having considerable potential as a tool for understanding disease pathogenesis and progression. Conventional imaging modalities like radiography are only able to detect structural changes that occur in the later stages of OA, whereas PET/MRI can potentially detect early disease-related changes and serve as a tool for monitoring the effect of new disease-modifying drugs at an early stage.

Studies looking at ^18^ F-FDG uptake in knee OA patients found a diffuse increase in uptake compared with healthy controls, indicating the presence of inflammation suggestive of synovitis, in particular affecting the medial aspect of the knee, where knee OA is usually most severe. 64 Prominent FDG uptake is also seen in the intercondylar notch and in the subchondral bone marrow areas corresponding to MRI-detected BMLs. 65 Another study found increased SUVmax in the central part of the knee joint reflective of synovitis of the synovial envelopes enclosing the cruciate ligaments centrally in patients with a painful knee, indicating a positive relationship between knee pain and ^18^ F-FDG uptake in knee OA. 66

**Fig. 8:**
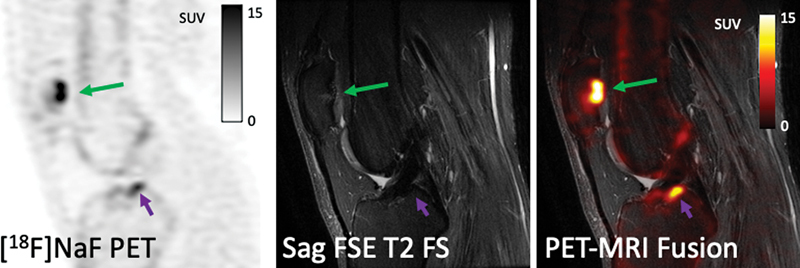
A 29-year-old man (body mass index: 26.3) with posttraumatic osteoarthritis (9 years after anterior cruciate ligament tear and reconstructive surgery). Heavily elevated NaF uptake is seen in the patella, correlating with cartilage loss and bone marrow lesion on magnetic resonance imaging (MRI) (green arrow). There is also a highly elevated uptake in the central tibia without any morphological correlate on MRI.

Increased subchondral bone remodeling has been implicated as a mechanism of OA progression, affecting not only the bone but the adjacent tissues as well, and bone activity imaging with ^18^ F-NaF PET was shown to correlate with histomorphometric assessment. 59 In patients with knee OA, significantly higher ^18^ F-NaF PET uptake was found in regions with subchondral BMLs, osteophytes, and sclerosis compared with regions of normal-appearing bone ( Fig. 8 ). 67 Interestingly, a large proportion (37%) of high uptake regions on ^18^ F-NaF PET did not correspond with abnormalities on MRI.

Similar results were found by Watkins et al, showing increased ^18^ F-NaF PET in both regions with and without structural findings in patients with knee OA compared with healthy controls. 68 These findings suggest that increased bone metabolism as detected by PET/MRI may be an early sign of OA, indicating changes in the subchondral bone before morphological changes are seen on MRI examination. This could be used as a biomarker for early OA. This hypothesis is further supported by the association between increased body mass index, a well-known risk factor for knee OA, and increased ^18^ F-FDG and ^18^ F-NaF uptake in the knees. 69 In patients with a unilateral reconstructed anterior cruciate ligament tear, a common knee injury that predisposes to posttraumatic OA, a significant increase of ^18^ F-NaF PET uptake was observed in the subchondral bone of the injured knee compared with the uninjured contralateral knee. A spatial relationship between bone and cartilage abnormalities was also observed in early OA, supporting the concept of studying whole-joint disease mechanisms in OA. 70

A more recent study found a correlation between SUVmax of ^18^ F-NaF PET in the subchondral bone with synovitis on contrast-enhanced MRI. Synovitis was more intense in areas adjacent to subchondral bone with high metabolic activity than in normal subchondral bone without ^18^ F-NaF uptake, 71 which is in line with the hypothesis that synovitis plays a role in the formation of osteophytes and subchondral bone alteration. 72 Preliminary data of PET/MRI of BMLs in patients with knee pain or previous knee injury showed high ^18^ F-NaF uptake at BML sites, whereas there was only negligibly increased ^18^ F-FDG uptake, 67 suggesting that bone remodeling plays a larger role in the development of BMLs than inflammation.

An acute response in bone physiology after bone loading can also be quantified using ^18^ F-NaF PET kinetics. An increase in SUVmax of up to 131% in subchondral bone tissue was found in healthy subjects after completion of an exercise protocol. 73 In a study of knee OA patients, large changes in subchondral bone ^18^ F-NaF PET uptake in regions with and without structural abnormalities were found after a squatting exercise. Most of these regions with focally high metabolic response to exercise showed structural progression of OA assessed using MOAKS at 2-year follow-up. 74 This suggests that ^18^ F-NaF PET after acute loading stress could be used to identify areas at high risk of OA progression, even before detectable structural abnormalities are seen.

##### Rheumatoid Arthritis

With the introduction of new targeted disease-modifying drugs for RA, sustained remission of disease can more frequently be achieved, and treatment strategies have shifted from symptom relief to prevention of structural damage and disability. 75 Early initiation of these therapies is crucial in maximizing long-term outcomes and, as a result, new imaging methods capable of detecting early inflammatory and cellular changes in RA are needed.

MRI allows for the detection of inflammation before the development of structural lesions visible on radiographs or computed tomography (CT). It has proven to be a powerful and efficient tool for detection and treatment monitoring in RA 76 and found to provide prognostic information. 77 ^18^ F-FDG PET offers an alternative quantitative and more sensitive method for evaluating inflammation in early RA, and ^18^ F-FDG uptake has been correlated with disease activity and C-reactive protein (CRP) levels in RA patients. 78 The feasibility of hybrid PET/MRI was demonstrated in early RA of the hand with areas of increased uptake of ^18^ F-FDG corresponding with sites of synovitis and tenosynovitis on CE-MRI. 79 Another study assessing anti-inflammatory treatment in 16 knees with active RA showed that synovial ^18^ F-FDG uptake correlated with CE-MRI inflammatory parameters and synovial thickness on ultrasonography as well as serum CRP and metalloproteinase-3 in both cross-sectional and longitudinal analysis. 80

In addition to ^18^ F-FDG, new PET tracers with new molecular targets may offer greater sensitivity for the detection and grading of inflammation. For example, promising results were reported for ^11^ C-Choline as a marker for cellular proliferation, 81 and macrophage tracers like ^11^ C-(R)-PK11195, 82 [ ^11^ C]DPA-713, 83 and 18F-fluoro-PEG-folate. 84

##### Juvenile Idiopathic Arthritis

A less extensively researched application for ^18^ F-FDG PET/MRI is children with JIA. One study found that PET/MRI showed more joints with inflammation than physical examination, but these additional findings did not correlate with any clinical characteristics. 85

##### Pain

Another field of research using PET/MRI is neuropathic pain secondary to nerve damage or nociceptive inflammatory pain. Although MRI is currently the main modality for high-resolution imaging of peripheral nerve abnormalities, it lacks specificity in detecting sources of neuropathic pain. Hybrid PET/MRI could help overcome this shortcoming by identifying nerve lesions through PET and high-resolution anatomical imaging through MRI at the same time. 86 PET imaging with ^18^ F-FDG is able to detect increased metabolic activity secondary to neuroinflammatory processes in painful nerve lesions correlating with pain symptoms. 87 Although mainly applied to the spine, the potential to detect nonspinal sources of pain was shown. 87

**Fig. 9:**
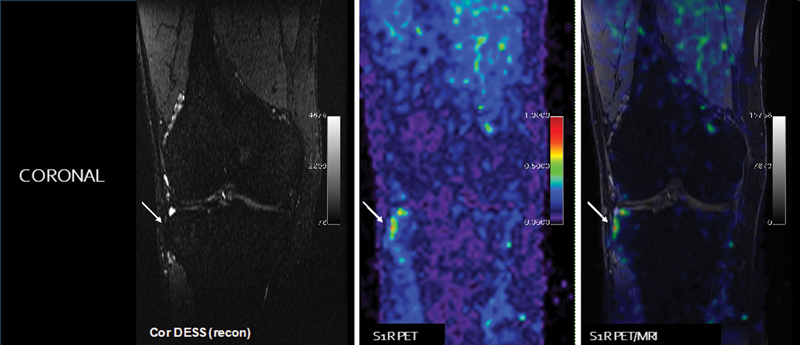
Young adult man with difficult to diagnose anterolateral knee pain > 1 year. Conventional knee magnetic resonance imaging (MRI) scans have been negative, and patient failed standard of care management until this point. Sigma-1 receptor positron emission tomography (PET)/MRI reveals increased radiotracer uptake at the insertion of the iliotibial band on Gerde's tubercle. This finally gave the referring physician good insight on how to move forward on what appears to be a traction enthesitis at this location. New therapeutic approaches, including local pain injections and physical therapy, led to eventual pain relief. (Courtesy of Sandip Biswal, MD, University of Wisconsin-Madison.)

Other notable tracers for neuropathic pain imaging include ^11^ C-PK11195 to image activated microglia and macrophages in neuroinflammation, 88 and 18F-FTC-146 to directly image pain signaling pathways by binding to sigma-1 receptors. 89 The potential of ^18^ F-FTC-146 was recently demonstrated in a case report of successful treatment of a patient with refractory knee pain caused by an undiagnosed intra-articular synovial lipoma. 90 Fig. 9 shows another example of the successful use of ^18^ F-FTC-146 to identify the source of pain.

## Conclusion

This review addresses the potential of perfusion and diffusion MRI and the combination of a variety of PET tracers with MRI for assessing inflammation and pain in the knee. These techniques have helped advance our understanding of inflammatory MSK disease, and they have opened up many new research opportunities. Each imaging technique has its own advantages and disadvantages and corresponding challenges for clinical implementation.
